# The impact of a computerized decision aid on empowering pregnant women for choosing vaginal versus cesarean section delivery: study protocol for a randomized controlled trial

**DOI:** 10.1186/s13063-015-1070-x

**Published:** 2015-12-03

**Authors:** Saeid Eslami, Azam Aslani, Fatemeh Tara, Leila Ghalichi, Fatemeh Erfanian, Ameen Abu-Hanna

**Affiliations:** Pharmaceutical Research Center, School of Pharmacy, Mashhad University of Medical Sciences, Mashhad, Iran; Department of Medical Informatics, Academic Medical Center, University of Amsterdam, Amsterdam, Netherlands; Department of Medical Informatics, Faculty of Medicine, Mashhad University of Medical Sciences, Mashhad, Iran; Department of Gynecology, Faculty of Medicine, Mashhad University of Medical Sciences, Mashhad, Iran; Mental Health Research Centre, Iran University of Medical Sciences, Tehran, Iran; Nursing and Midwifery School, Mashhad University of Medical Sciences, Mashhad, Iran

**Keywords:** Decision support, decision aid, vaginal birth, caesarean section, prenatal education, public health informatics, patient empowerment

## Abstract

**Background:**

Cesarean delivery on maternal request (CDMR) is one of the main reasons for cesarean delivery in Iran, and women often need help in making a decision about the delivery options available to them. The main objective of this study is to evaluate the effect of a computerized decision aid (CDA) system on empowering pregnant women in choosing an appropriate mode of delivery. This CDA contrasts the advantages and disadvantages of vaginal versus cesarean section delivery in terms of their value to the individual woman.

**Methods/Design:**

The protocol concerns a randomized trial study that will be performed among Iranian women. Four hundred pregnant women will be recruited from two private and two public prenatal centers in Mashhad, Iran. They will be randomly assigned to either an intervention or a control group. The designed CDA will be provided to the intervention group, whereas the control group will only receive routine care. The CDA provides educational contents as well as some recommendations. The CDA’s knowledge base is obtained from the results of studies on predictors of cesarean delivery. The CDA’s software will be installed on women’s computers for use at home.

The two primary outcomes for the study are O’Connor’s Decisional Conflict Scale and knowledge as measured by true/false questions. Actual mode of delivery (vaginal versus cesarean) will be compared in the two groups.

**Discussion:**

We investigate the effect of a CDA on empowering pregnant women in terms of reducing their decisional conflict as well as on improving their clinical knowledge pertaining to mode of delivery.

**Trial registration:**

This trial is registered with the Iran Trial Registrar under registration number IRCT2015093010777N4 and registration date 26 October 2015.

## Background

Caesarian section rate in Iran is three times the global average rate. According to a report released by the Iranian Ministry of Health and Medical Education in 2005, caesarian sections constitute 40.7 % of all deliveries in Iran, including 52 % of the deliveries in Tehran (the capital of Iran), of which 64 % are performed in private hospitals [1]. Over the past 30 years, the rate of caesarian sections has been increased in a referral hospital in Tehran from 14.3 % in 1979 to 22.7 % in 1989, 52.5 % in 1999, and 85.3 % in 2009 [2].

A cross-sectional study on 824 pregnant women in Tehran, reported that 72 % of pregnancies with cesarean section were elective. Of these elective pregnancies, 22 % were performed because of a maternal request. Among pregnant women with these requests, 71 % had no explanation for their request. In addition, 65 % of doctors suggested cesarean delivery for their patients without a true medical indication [3]. Results of a study with 350 questionnaires filled out by pregnant women at waiting time for their prenatal visit in eight public health centers and four private offices in Mashhad, Iran, showed that 90 of these pregnant women (26 %) preferred cesarean delivery when a vaginal delivery was appropriate (unpublished observation). These findings are corroborated by studies showing that 70 % of pregnant women in Iran request cesarean section for reasons unrelated to health needs, such as physician's recommendation, personal request, and compliance to the spouse’s preference [4, 5].

Maternal request is one of the main reasons for increasing the rate of cesarean section in Iran. Cesarean delivery on maternal request (CDMR) is defined as the event in which a pregnant woman prefers and requests a cesarean delivery, without maternal or fetal indication, rather than proceeding with a plan for vaginal delivery [6].

The great variance in CDMR across countries suggests that women need help making a decision about the mode of delivery [6–8]. Gynecologists, midwives, and also the family, have significant effect on pregnant women’s decision on mode of delivery [7, 8]. As a result, the decision-making process may be hindered, for example, a disagreement between a physician and the family (unpublished observation).

Despite the importance of involving women in the decision-making process, multiple studies demonstrated that many women have inadequate knowledge to make informed decisions about their pregnancies and maternity cares [3, 8, 9]. Appropriate information and support can provide women with confidence to make their decision, and ultimately, the power to own and justify the decision that they had made. Having the confidence to ask questions, understand clinical information, and communicate delivery mode preferences to the health care professionals, were also considered important factors in making a firm decision by pregnant women [10].

Decision-making is the process of choosing between alternative courses of action (in this study, vaginal delivery versus CDMR). Decision aids (DAs) are increasingly being developed to help patients make informed decisions. Most of the decision aids used in obstetric care are in the form of paper-based information booklet or leaflet [11–15], audio book, or audiotape with an illustrated booklet [16]. Computer-based DAs have also been used in the format of videodiscs [17], CD-ROM programs [18, 19], and Internet sites [20, 21]. Computerized DAs (CDAs) belong to a different group of DAs due to the way they present information that is tailored for each individual [22]. Such aids also reinforce comprehension in a consistent way by providing feedback from the user to the system [23] and using graphic-numeric and text-anchored formats [24].

Some studies have shown evidence supporting the effect of computerized decision aids on improving knowledge of patients and decreasing their decisional conflict [22, 23]. We use the decisional conflict scale (DCS) to measure patients’ perceptions of their uncertainty in choosing a care option, factors contributing to this uncertainty, and effectiveness of decision-making [25].

Decision aids may enhance decision-making by providing a systematic approach to decision-making and enhancing patient autonomy [26, 27]. Specifically, we expect the CDA to improve effective decision-making in pregnant women by clarifying risks and benefits of vaginal versus cesarean delivery, while also considering pregnant women's personal values and their care. One need not expect this improvement to have a negative impact on anxiety.

According to Orem's theory [28], decision-making constitutes a first phase of “deliberative self-care.” People who are competent at self-care and understand entirely the courses of actions are usually open to these actions and receptive to their effectiveness. Hence, improving the decision-making process by decision aids has the potential to enhance self-care. Informed decision-making can potentially reduce the over-use of expensive surgical procedures [29].

The objective of our overall project is to design, construct and evaluate a decision aid to help pregnant women choose mode of delivery. The system’s knowledge-base is obtained from the literature on cesarean delivery predictors. Our hypothesis in this study is that decision aid decreases decisional conflict and increases patient knowledge about cesarean and vaginal delivery modes. Decrease in the frequency of cesarean delivery on maternal request is a secondary expected outcome.

## Methods/design

### Design of the intervention

We developed a decision aid for the mode of delivery using the three steps of the Ottawa Decision Support framework: identifying needs, providing decision support and evaluating decision support. A cross sectional study was done to reveal pregnant women’s preferences and concerns about childbirth. We specified the variables associated with cesarean delivery in pregnant women based on a literature study and a field study (under publication). The need for a decision aid was identified following another literature review and by forming focus groups with experts.

Based on the identified needs in the first step, we developed a computerized decision aid (CDA) system. This computer-based decision support system is an application that pregnant women can use alone or with their families. The program will be installed on a personal computer and/or laptop of pregnant women who are recruited for the intervention group, to be used at home. Capabilities of the installed software include the following:

1. Information retrieval: A list is available of topics that contain information about the types of delivery. Hyperlinks to educational content are also placed in the decision-support section. The included information explains the tradeoffs between vaginal delivery versus CDMR.
2. Clinical data entry: Two categories of information can be entered into the computer by pregnant women. These are listed below.
  - General information (for example, age, gestational age, weight, and height) and health history (for example, parity, history of smoking, drug abuse, and anxiety).
  - Response to 15 statements organized in two categories pertaining to 1) possible reasons for seeking CDMR, such as desire to plan or avoid unplanned cesarean section, and 2) factors that may encourage the selection of natural delivery, such as not requiring anesthesia, surgery or shorter rest after delivery.
  - Decision-support tool: this is an interactive computerized module for supporting the selection of delivery mode. Women are given information about the outcomes associated with vaginal delivery, elective caesarean section, and emergency caesarean section. They can include their personal preferences for possible outcomes by answering questions about 15 statements (section b. clinical data entry). The software program combines the answers and represents the overall results about vaginal delivery versus cesarean section. We also provide an interactive self-assessment and feedback module to educate and correct their misunderstandings about delivery modes.

### Ethics

The Ethical committee of Mashhad University of Medical Sciences has granted ethics approval to conduct this trial (326955, 30^th^ August 2015). A signed consent form is obtained from all participants in this trial at the time of recruitment.

### Setting for the trial

The study will be conducted in the primary health care setting of Mashhad, the second largest city in Iran with approximately three million residents. All women who plan to give birth at the two public health care centers and the two private offices can participate in this study if they sign the consent form. The selection of these facilities is based on their readiness for project implementation. Six gynecologists are practicing at the two public health care centers offices, and they see about 1,700 pregnant women per month (250 to 300 returning patients). The two gynecologists in the private offices see about 700 pregnant women per month (100 to 150 returning patients).

### Study design

This study follows is a randomized controlled trial design. Subjects (pregnant women) are the unit of randomization; they are randomly allocated to each arm of the study. Both the intervention and control group will receive usual care, but the intervention group will also receive the CDA. An independent researcher will centrally perform the randomization by using the computer program on www.randomization.com. Block randomization will be applied to ensure equal group sizes within each center. Because the study is not blinded, we use blocked randomization with randomly selected block sizes of two and four. For each center, the independent researcher will prepare opaque, sequentially numbered, and sealed coded envelopes, with a note for either the decision aid use or not. At each center, the clinic secretary will check eligibility of each pregnant woman visiting the clinic and, if eligible, will provide her with an explanation of the aims and scope of the study and will orally invite her to participate in the study. If the invitee accepts the invitation, then she will be asked to sign the informed consent form. Then the secretary writes the woman’s name and phone number on the next closed envelope. At the end of the day, all envelopes, unopened as of yet, are collected by a research assistant. At the university, this researcher assistant opens the envelopes and allocates the participants of that day to the decision aid or control group. After group allocation, the research assistant will call the participants by phone. Those in the intervention arm will be invited to a software educational session (consisting of 10 women in total) and invited to fill in the paper questionnaires. Those in the control arm will be invited to only fill in the questionnaires. All participants (in the intervention and control arms) will be invited to fill in the questionnaires again two weeks before the expected date of delivery. Blinding of researchers and women is not practical in this study, but the gynecologists are unaware of the patient allocations.

The duration of the intervention is considered from recruitment to the time of delivery. The evaluation of the intervention will be performed at 38 weeks of gestation and before delivery. We will report all cases due to premature delivery.

### Sample size

We aim to detect absolute decrease of 15 % in the rate of decisional conflict between women in the intervention and control groups. A sample size of 400 pregnant women (200 per trial arm) is needed to detect this difference with a power of 80 % and 5 % risk of type I error. We used O’Connor’s Decisional Conflict Scaling method (0 to 100) to measure decisional conflict (or insecure feeling). The anticipated difference in outcome measure was based on a study assessing decisional conflict based on O’Connor's Decisional Conflict Scale of 212 Iranian pregnant women (mean 33.6; SD = 17.6) [8]. According to the O’Connor’s Scaling Method, decisional-aid tools are most effective when used for subjects with a score of 35 and above [25].

### Inclusion criteria

Pregnant women 16 years and older who had 28 or more weeks of gestation with a singleton pregnancy and who were seen by a participating gynecologist are eligible to be enrolled in the study. We include women who see only their own gynecologist in both the private office and public health centers during pregnancy.

### Exclusion criteria

Women are excluded if they do not provide a written informed consent, are non-Persian speaking, unable to read and write, or unable to work with a computer. Women whose method of delivery has been predetermined by the physician, based on their clinical condition, are also excluded. In addition, women with miscarriage or other complications are excluded from the study in any group.

### Data collection

Women are asked to complete two questionnaires: the first one upon entering the study and the second one during their 38^th^ week of gestation. Other information (related to women's health status) will be collected from pregnant women’s medical records in the hospitals and clinics.

### Outcome

The two primary outcomes for the study are the mean of overall decisional conflict and knowledge scores. The decisional conflict will be measured by O’Connor Decisional Conflict Scale (DCS) [25, 30]. The DCS subscales are secondary outcomes, and they will be compared between the two groups of women. The results of this study will show the specific three affected subscales in the pregnant women's decisional conflict scale. Subscale 1 addresses the uncertainty about choosing among alternatives. Subscale 2 addresses factors contributing to uncertainty, including a - feeling uninformed about the alternatives, benefits and risks; b - being unclear about personal value; c - feeling unsupported in making a choice or pressured to choose on course of action. Finally, subscale 3 addresses the perceived effectiveness of the decision [30].

The DCS scale is assessed for validity and reliability using statistical tests, including factor analysis and Cronbach alpha test. A Persian version of the scale was used in 212 pregnant women in Mashhad, Iran.

Knowledge acquisition will be evaluated by 15 true/false questions that are provided by the researchers. The actual mode of delivery, which will be measured by data obtained from hospital records after the intervention. We will determine the studied pregnant women's decision on mode of delivery before and after intervention and compare the results with the actual delivery mode. We will not evaluate cases with emergency delivery but report on them in the two groups.

The acceptability of the CDA by the enrolled pregnant women in the intervention arm will be assessed by interviewing them and analyzing open-ended comments. Our participants' experiences regarding the use of the decision aids will be reported. Also we will discuss our experience with the CDA’s implementation.

### Statistical analysis

Differences in the primary outcomes will be analyzed using the “intention-to-treat” principle. The mean decisional conflict score and the mean knowledge score will be compared, using the t-test, to the respective means in the control group. Comparing the actual mode of delivery in the intervention and control groups will be based on hospital maternity records and differences will be tested by the Chi square test. We will calculate the Pearson correlation coefficient between the score of decisional conflict and the mode of delivery.

We will handle missing data by a multiple imputation approach. The multiple imputation procedure replaces each missing value with a set of acceptable values that represent the uncertainty about the right value to impute. These multiply imputed data sets are then analyzed by using standard procedures for complete data and then the results from these analyses are combined.

## Discussion

This study will investigate the effect of a CDA in decisional conflict of pregnant women and their knowledge acquisition pertaining to mode of delivery. This system aims to inform pregnant women and to support them in making decisions about the delivery method by addressing their concerns about the upcoming delivery. We hypothesize the CDA can decrease pregnant women’s decisional conflict and increase their knowledge about the delivery methods. We expect a 15 % effect size in the decisional conflict scale and improvement in the knowledge due to the intervention. We expect the requests for CDMR to decline due to the information and knowledge pregnant women receive through the decision aid tool provided in the study. We will investigate the difference in the DCS subscales, especially the change in the effective decision subscale, and report on that. We will also discuss the difference between the stated pregnant women's preference on mode of delivery with the actual delivery.

Some studies (McCourt [31], Mazzoni 2010 [32], and Weaver [33]) reported that only a minority of women preferred caesarean delivery, particularly in the absence of clinical indicators. We will compare our findings with these reports and discuss possible differences [31–33].

We based our work on the Ottawa Decision Support Framework (ODSF). According to ODSF, patients can be guided through multiple phases in making an informed decision regarding their social or health status. During this process, decision-support requirements are identified, designed, and evaluated. This theory suggests that women would benefit most from decision-aids that encourage a thoughtful and deliberative process. Ideally, this process should (1) increase women’s awareness of the risks and benefits of each delivery methods, (2) explain how to increase the accuracy perception of their risk, (3) assist them in evaluating the risks and benefits in the context of their personal preferences, and (4) encourage women to discuss their concerns with a health care provider [17].

There are various threats to this trial. We are aware of the perceived barriers to implementation of computer-based decision aids for women including environmental factors (time pressure, cost, and access), computer issues (like computer literacy and troubleshooting), and factors pertaining to people’s preferences and biases (like women’s prior delivery preferences and clinician preference). In addition there are some concerns that the CDA would only help women who were already informed, interested, and eager to be involved in their care [34]. Although this might mean the intervention is especially effective in subgroups of pregnant women, the randomization should take care of selection bias. Finally, there is the risk, although low, of contamination at the level of the participants by women in the intervention group sharing the decision aid with women in the control group, or on the gynecologist level, by transferring information to the women in the control group. We will tolerate this level of contamination to avoid complexity and cost of the study.

### Trial status

The CDA prototype had already been completed in July 2014, and thereafter, a pilot period of 6 months ensued to identify any issues requiring improvement.

## Abbreviations

CDMR: cesarean delivery on maternal request
DAs: decision aids
CDA: computerized decision aid
DCS: decisional conflict scale
